# Isolation of Four Lytic Phages Infecting *Klebsiella pneumoniae* K22 Clinical Isolates from Spain

**DOI:** 10.3390/ijms21020425

**Published:** 2020-01-09

**Authors:** Pilar Domingo-Calap, Beatriz Beamud, Justine Vienne, Fernando González-Candelas, Rafael Sanjuán

**Affiliations:** 1 Department of Genetics, Institute for Integrative Systems Biology, I^2^SysBio, Universitat de València, Universitat de València-CSIC, 46980 Paterna, Spain; 2 Institute for Integrative Systems Biology, I^2^SysBio, FISABIO-Salud Pública, Generalitat Valenciana, Universitat de València-CSIC, 46980 Paterna, Spain; beatriz.beamud@uv.es; 3 College West Flanders, Howest University, 8500 Kortrijk, Belgium; justine.vienne@telenet.be; 4 FISABIO-Salud Pública, Generalitat Valenciana, and CIBER in Epidemiology and Public Health, Institute for Integrative Systems Biology, I^2^SysBio, Universitat de València-CSIC, 46980 Paterna, Spain; fernando.gonzalez@uv.es; 5 Institute for Integrative Systems Biology, I^2^SysBio, Universitat de València-CSIC, 46980 Paterna, Spain; rafael.sanjuan@uv.es

**Keywords:** *Klebsiella pneumoniae*, bacteriophage, phage therapy

## Abstract

The emergence of multi-drug-resistant bacteria represents a major public-health threat. Phages constitute a promising alternative to chemical antibiotics due to their high host specificity, abundance in nature, and evolvability. However, phage host specificity means that highly diverse bacterial species are particularly difficult to target for phage therapy. This is the case of *Klebsiella pneumoniae*, which presents a hypervariable extracellular matrix capsule exhibiting dozens of variants. Here, we report four novel phages infecting *K. pneumoniae* capsular type K22 which were isolated from environmental samples in Valencia, Spain. Full genome sequencing showed that these phages belong to the *Podoviridae* family and encode putative depolymerases that allow digestion of specific K22 *K. pneumoniae* capsules. Our results confirm the capsular type-specificity of *K. pneumoniae* phages, as indicated by their narrow infectivity in a panel of *K. pneumoniae* clinical isolates. Nonetheless, this work represents a step forward in the characterization of phage diversity, which may culminate in the future use of large panels of phages for typing and/or for combating multi-drug-resistant *K. pneumoniae.*

## 1. Introduction

The emergence of multi-drug-resistant bacterial strains as a result of indiscriminate antibiotic use constitutes a major public-health issue worldwide. Alternative treatments are needed to combat these pathogenic bacteria. Phages (viruses that infect bacteria) offer a promising tool to lyse pathogenic bacteria without depleting other species of the microbiome, avoiding dysbiosis. However, their high specificity also poses a difficulty for the development of a curative treatment, since fast bacterial typing (not always feasible) or very large phage panels are needed to combat highly diverse bacteria [1]. For this reason, the use of phage cocktails has been suggested, but this requires a comprehensive analysis of the interactions among cocktail components. Phage cocktails have been already tested in animals and in a few clinical cases, mainly as compassionate treatments, with encouraging results [2].

Among multi-drug-resistant bacteria, nosocomial infections probably represent the greatest current challenge, particularly the ESKAPE group constituted by *Enterococcus faecium*, *Staphylococcus aureus*, *Klebsiella pneumoniae*, *Acinetobacter baumannii*, *Pseudomonas aeruginosa* and *Enterobacter species* [3]. Within this group, carbapenem-resistant *Klebsiella pneumoniae* is considered as a WHO priority. *K. pneumoniae* is a Gram-negative encapsulated bacterium that causes opportunistic infections and exhibits a wide ecological distribution. *Klebsiella* can be isolated from soils as a free-living organism and can be found in the normal flora of the mouth, skin, and intestines [4]. In some cases, *K. pneumoniae* can cause pathogenic infections such as pneumonia, urinary tract complications, cholecystitis, diarrhea, osteomyelitis, meningitis, and sepsis. Unfortunately, *K. pneumoniae* shows high antimicrobial resistance gene diversity, making successful treatment difficult. *K. pneumoniae* is intrinsically resistant to ampicillin due the presence of penicillinase in its chromosome, and many additional resistances are encoded in plasmids acquired via horizontal gene transfer [5]. Many *K. pneumoniae* strains have been found to be resistant to third-generation cephalosporines, fluoroquinolones, carbapenems and colistin [6]. A recent study involving 244 hospitals from 32 countries as part of the European Survey of Carbapenemase-Producing *Enterobacteriaceae* showed that the propensity of *K. pneumoniae* to spread in hospital environments correlates with the degree of resistance, and that carbapenemase-positive isolates are those exhibiting the highest transmissibility [7].

The *K. pneumoniae* exopolysaccharide (EPS) capsule is a virulence determinant and can be used as an epidemiological marker [8]. Importantly, phage infection has been shown to be at least partially capsular type-dependent [9], although this conclusion is preliminary given the relatively small number of *K. pneumoniae* phages tested so far. Previous studies have shown that most *K. pneumoniae* phages encode depolymerases, enzymes capable of digesting the *Klebsiella* capsule. Although most of these phage genomes encode for just one depolymerase, some have been shown to encode multiple depolymerases, allowing them to infect multiple capsular types. To date, the broadest-range *Klebsiella* phage described, *ØK64-1*, contains nine functional capsule depolymerase-encoding genes [10]. Characterizing the diversity of *Klebsiella* phages is of special interest in order to obtain more potent and broader-range anti-*Klebsiella* therapies.

Here, we describe and characterize four novel *Klebsiella* phages isolated from environmental samples in Valencia (Spain). Our results support previous data showing that *Klebsiella* phages are highly capsular type-specific, which represents a significant obstacle for the development of phage treatments against multi-drug-resistant *Klebsiella* sp. Albeit highly specific, the reported phages exhibit strong lytic activity and infect a previously untargeted *Klebsiella* capsular type.

## 2. Results

### 2.1. Isolation and Lytic Activity of Klebsiella pneumoniae Phages.

**Figure 1:**
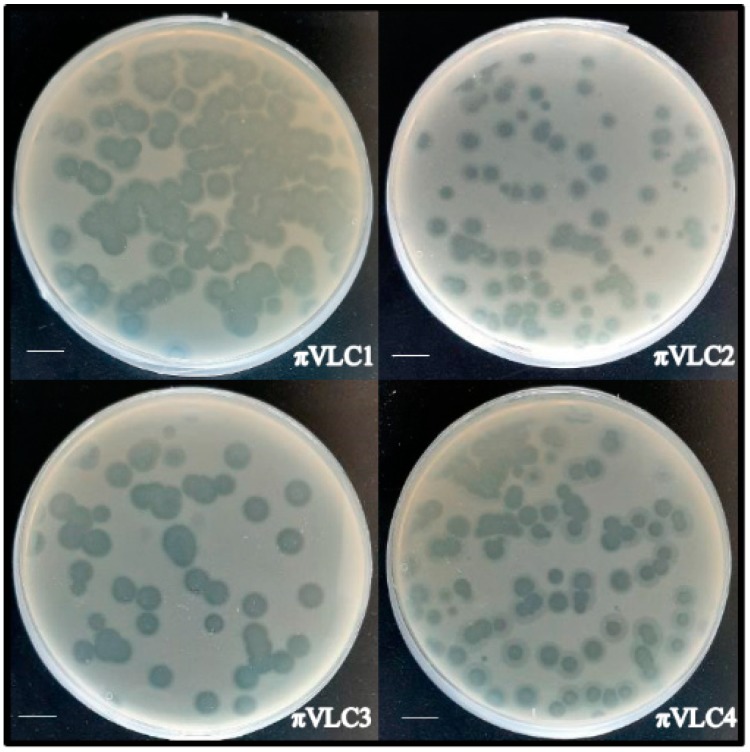
Plaque assays of the four novel *K. pneumoniae* phages. Plaques were allowed to develop in soft agar media overnight at 37 °C. Scale bar: 1 cm.

**Figure 2:**
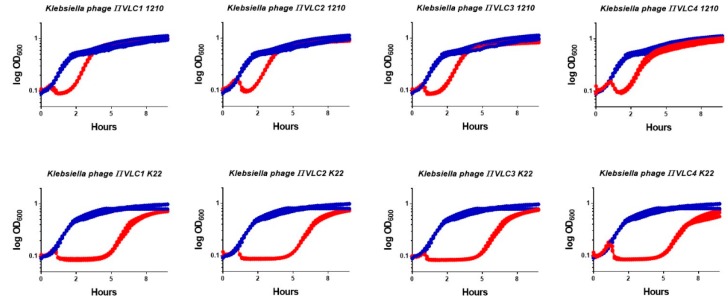
Lysis assays. Optical density of cultures of *K. pneumoniae* belonging to isolate *1210* (top) or the *K. pneumoniae K22* reference strain (bottom). Cultures were inoculated with 10^5^ PFU. Red: three replicate assays with phage. Blue: three replicate assays of non-infected control.

Four novel phages infecting *K. pneumoniae*, named *πVLC1* to *πVLC4*, were isolated from irrigation ditches and soil near sewage water plants in Valencia, Spain (Table 1). Samples were filtered and tested on soft agar semi-solidified media containing a lawn of a clinical isolate (*1210*) of *K. pneumoniae* belonging to capsular type K22. To isolate each phage, a triple plaque-to-plaque transfer was carried out. Subsequently, each isolated plaque was used to infect log-phase *K. pneumoniae* 1210 cultures, and the supernatant was titrated by the standard plaque assay. Titers after this amplification step ranged from 1 × 10^10^ to 3 × 10^11^ plaque forming units (PFU) per mL (Table 1). Each phage exhibited a different plaque morphology based on plaque size, turbidity, and presence of a surrounding halo (Figure 1), suggesting that each was a different phage. To assess lytic activity, log-phase *K. pneumoniae* 1210 cultures were inoculated with 10^5^ PFU of phage and changes in OD_600_ were measured (Figure 2). In addition, we used the *K. pneumoniae K22* reference strain obtained from the Statens Serum Institute (Copenhagen, Denmark) to test whether our phages infected other K22 isolates. These assays revealed a strong lytic effect, as also suggested by the large and clear plaques formed in bacterial lawns.

### 2.2. Transmission Electron Microscopy

**Figure 3:**
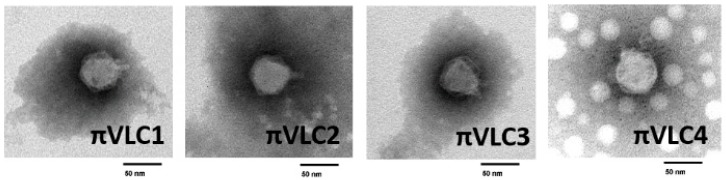
Transmission electron micrographs of the four novel *K. pneumoniae* phages. Scale bar: 50 nm.

Transmission electron micrographs were obtained directly from phage-lysed *1210* liquid cultures showing approximately >10^10^ PFU/mL. All phages were morphologically similar and exhibited typical features of the *Podoviridae* family (*Caudovirales* order) including an icosahedral head with a diameter on the order of 50 nm and a short tail of 10–20 nm (Figure 3).

### 2.3. Genome Sequencing and Sequence Analysis of the Four Novel Klebsiella pneumoniae Phages

The four phages infecting *K. pneumoniae* were fully sequenced using the MiSeq Illumina platform. The sequencing run yielded approximately 180,000 paired-end reads for *Klebsiella* phages *πVLC1*, *πVLC2* and *πVLC4* and 63,000 paired-end reads for phage *πVLC3*. High coverage allowed us to rapidly assemble the reads from each sample into a single contig. Genome sizes and GC contents were very similar, ranging between 43.3 and 44.7 kb and between 53.75% and 53.96%, respectively. Putative coding sequences (CDSs) found in the four genomes varied from 56 in phage *πVLC3* to 62 in phage *πVLC4*. High pairwise nucleotide identities (from 93.5% to 99.8%) were also observed (Table 2).

Sequence analysis confirmed that the four phages belonged to the *Podoviridae* family. BLAST analysis identified these *Klebsiella* phages as belonging to the genus *Drulisvirus*. To further check this, a multiple sequence alignment of all publicly available *drulisviruses* (*n* = 9), and two phylogenetically related phages (*LIMElight* and *VP93*) was performed. *Klebsiella* phages *πVLC1-4* were found to be collinear and highly conserved compared to the rest of *drulisviruses*, and distantly related to phages *LIMElight* and *VP93*, as expected. Specifically, average nucleotide identity (ANI) values between the isolated *Klebsiella* phages *πVLC* and the other *Drulisviruses* ranged from 86.04% to 90.88%, whereas they dropped to 60% when compared with *LIMElight* and *VP93*. The closest previously characterized bacteriophage, according to ANI values, was *Klebsiella* phage *vB_KpnP_SU552A*, isolated in Sweden.

**Figure 4:**
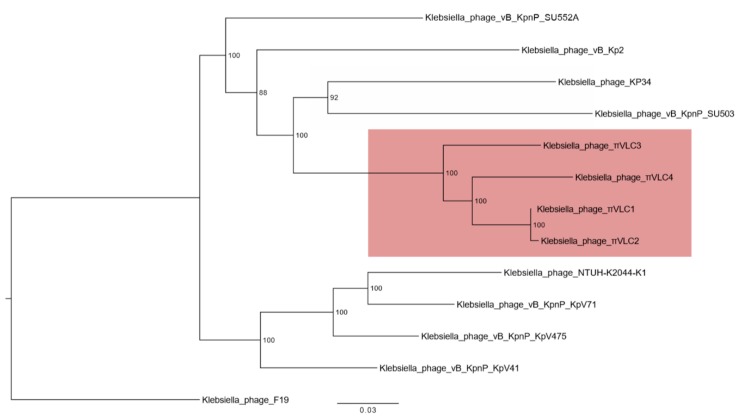
Maximum likelihood phylogenetic tree of whole-genome *Drulisvirus* sequences. Numbers indicate bootstrap values (1000 pseudo-replicates). The four phages isolated and characterized here are indicated in red.

A multiple whole-genome sequence alignment of *Drulisvirus* and *Klebsiella* phages *πVLC1-4* (58,184 positions) was used to obtain a maximum likelihood phylogeny. This indicated that phages *πVLC1-4* formed a monophyletic group with a high bootstrap support (Figure 4).

### 2.4. Functional Annotation of Klebsiella pneumoniae Phages πVLC1-4

**Figure 5:**
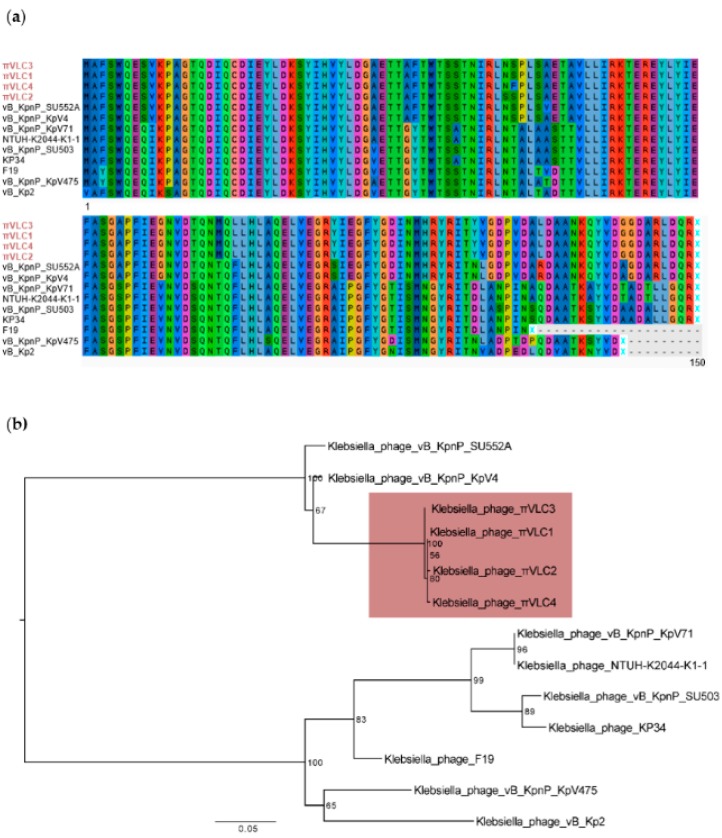
Tail fiber conserved domain alignment and phylogenetic relationships in the genus *Drulisvirus*. (**a**) Amino acid multiple sequence alignment of the conserved tail fiber protein domain. The four phages isolated and characterized here are indicated in red. Colors in the figure represent different amino acids. (**b**) Nucleotide maximum likelihood tree of the conserved tail fiber protein domain of the genus *Drulisvirus*. Numbers indicate bootstrap values (1000 pseudo-replicates). The four phages isolated and characterized here are indicated in red.

We found that all four phages encoded their own DNA and RNA polymerases and a lysis cassette composed of holin, endolysin and spanin. Importantly, for possible phage therapy applications, no signal of lysogeny was found in any of the *Klebsiella* phages described here. Interestingly, there were four genes encoding a tail fiber, a homing endonuclease and two hypothetical proteins with no orthologs among *Drulisvirus* phages. The tail fiber gene (ORF68, Table S1) exhibited high similarity with the EPS depolymerase of *Pantoea* bacteriophage *LIMElight*, with e-values of 1 × 10^−39^ and 75% identity in BLAST. This translated ORF (745 aa) was subjected to InterProScan for domain prediction and we found two significant matches. First, amino-acid positions 13–140 matched with the bacteriophage *T7* tail fiber protein IPR005604 (e-value of 2.7 × 10^−11^). This domain was highly conserved among *drulisviruses*. Phylogenetic analysis of the conserved tail fiber domain showed that the sequences belonging to *Klebsiella* phages *πVLC1-4* formed a well-supported cluster differentiated from other *drulisviruses* and related phages (Figure 5). Second, positions 308–610 matched with a pectin lyase domain (IPR012334, e-value of 4.8 × 10^−10^), supporting the enzymatic activity of this region. Hpred analyses revealed that the protein structure of this ORF has high similarity (probability of 100%, e-value of 3.9 × 10^−33^) with the bacteriophage *phiAB6* tailspike (5JSD_C). Therefore, we concluded that the depolymerase activity is probably contained in the phage tailspikes, despite the initial annotation as a tail fiber.

**Figure 6:**
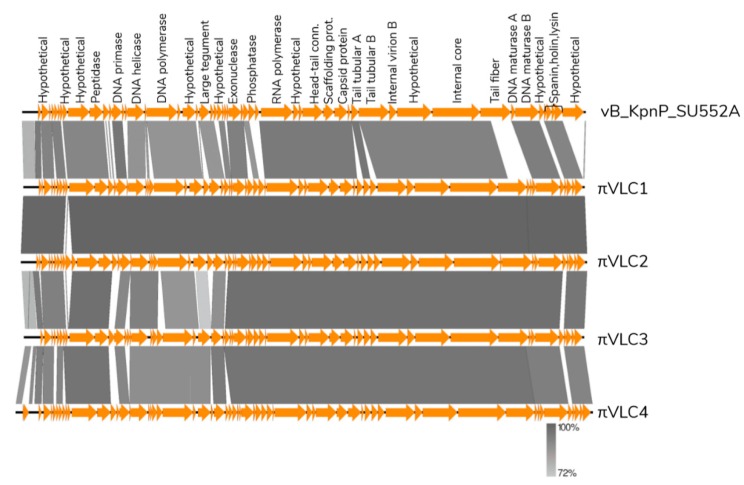
Genome comparison of *Klebsiella* phages *πVLC1-4* and the previously characterized *Klebsiella* phage *vB_KpnP_SU552A* (closest genome by ANI values) with EasyFig [11]. The arrows indicate the product encoded in each CDS, omitting small CDS to facilitate visualization.

Minor differences were found between the 45 shared orthologous genes encoded by phages *πVLC1-4* (Table S1). Differences resided in a few hypothetical proteins, homing endonucleases, and tail fibers (Figure 6). *Klebsiella* phages *πVLC1* and *πVLC2* were found to be highly similar, differing by 158 SNPs and 635 indels. The largest indel covered 485 nt and mapped to a 763 nt hypervariable region encoding a hypothetical protein. This region also contained 150 out of 158 SNPs identified, and corresponded to genome positions 3223–3992 for *πVLC1* and 3224–3492 for *πVLC2*. In addition, we found that *πVLC2* encoded a homing endonuclease not present in *πVLC1*, suggesting that although they are closely related phages, they should be considered as different phages.

### 2.5. Plaque Halos of Klebsiella pneumoniae Phages πVLC1-4

**Figure 7:**
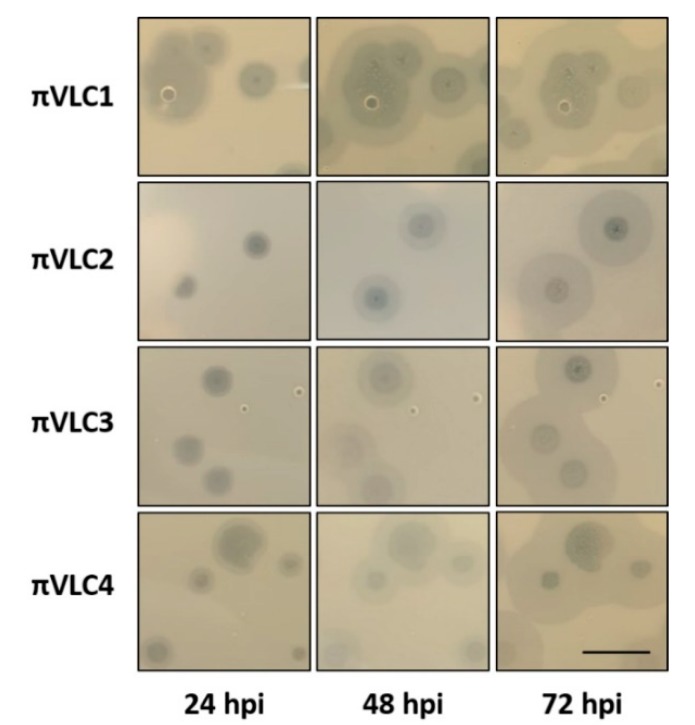
Development of plaque halos around *Klebsiella pneumoniae* phages *πVLC1-4* plaques. Notice that halos kept expanding after the central lysis area ceased to grow. Scale bar: 1 cm.

As showed by their genomic characterization, all four *Klebsiella* phages *πVLC1-4* encode for one depolymerase. Although not fully conclusive, the formation of plaque halos that diffuse around the lysis area suggests the presence of functional depolymerases capable of digesting bacterial capsules. Plaque images taken every 24 h for 3 days revealed that halos increased in size with time even when lysis did not proceed further (Figure 7). As suggested previously, the growing halos may contain non-infected stationary-phase cells whose optical density was reduced by the action of diffusing depolymerases on EPS capsules [12]. However, further microscopical and molecular characterization is needed to validate this observation.

### 2.6. Determination of Klebsiella pneumoniae Phages πVLC1-4 Host Range

To determine the host range of the newly discovered phages, we carried out spot tests in soft agar plates for a battery of 24 *K. pneumoniae* multi-drug-resistant clinical isolates obtained from local hospitals and belonging to 21 different capsular types. All phages produced plaques exclusively in *K. pneumoniae* K22, including the clinical isolate used for isolating the phage and another isolate from the same patient, in addition to the *K22* reference strain. We also found that the lytic activity of the four phages, as determined by OD_600_ measurements in liquid cultures, was restricted to these same *K. pneumoniae* K22 isolates.

## 3. Discussion

Understanding phage diversity in nature may help in the future development of phage-based treatments [13]. Although previous *Klebsiella* phages have been reported, this work provides the first isolation of *Klebsiella* phages from environmental samples in Spain and of *Klebsiella* phages infecting capsular type K22. Sequencing and virion morphology showed that the four *Klebsiella* phages were highly related, but different. Our reason for choosing K22 for phage hunting was solely based on strain availability, and although this is not considered as a major capsular type, it is not uncommon among clinical isolates [8]. The fact that we successfully isolated multiple phages infecting this strain suggests that it might possible to obtain a panel of phages capable of infecting a large variety of capsular types. The *Drulisvirus* genus contains nine published *Klebsiella* phage genomes: *F19* (NC_023567), *KP34* (NC_013649) [14], *Kp2* (NC_028664), *KpV41* (NC_028670), *KpV475* (NC_031025), *KpV71* (NC_031246), *NTUH*-*K2044*-*K1-1* (NC_025418), *vB_KpnP_SU503* (NC_028816) [15] and *vB_KpnP_SU552A* (NC_028870) [15]. *Klebsiella* phage *NTUH-K2044-K1-1* has been characterized in terms of host range and shown to selectively infect capsular type K1 [16]. Similarly, phages *KpV41*, *KpV475* and *KpV71* were found to have lytic activity mainly against K1 [17]. Bacterial mutants lacking the capsular EPS showed resistance to this phage, suggesting that host recognition factors are located in the capsule. As suggested by these authors, *Klebsiella* phage *NTUH-K2044-K1-1* and other phages could be used as a diagnosis tool or to treat *K. pneumoniae* K1 infections. Similar applications can be thought of for phages *πVLC1-4* for capsular type K22, as well as for other (undiscovered) phages and capsular types. In future work, it would also be interesting to test whether a combination of phages could be used to prevent the emergence of phage-resistant bacteria. This should depend on phage receptor usage, among other factors.

Our results suggest that the newly reported phages encode highly active depolymerases, based on the large halos observed in bacterial lawns after 72 h and on the confirmation of a pectin lyase domain. Structural sequence analysis predicted that the depolymerase activity of these phages resides in tailspikes. Tail fibers and tailspikes are responsible of specific recognition of bacterial surfaces [18], and depolymerases have been shown to participate in this process [19]. The fact that all four phages shared similar depolymerases may explain why they infect the same capsular type. Nonetheless, further studies including more capsular types, but also more K22 isolates, are needed to verify the generality of capsular type-dependency. It is noteworthy that the depolymerase of *Klebsiella* phage *NTUH-K2044-K1-1* has a sequence similar to those of *πVLC1-4* but different capsular type-specificity. Our results also suggest the potential use of depolymerases as enzybiotics [20]. Isolation of depolymerases and their use against *Klebsiella* biofilms can be a promising alternative in the fight against nosocomial bacteria in clinical environments and clinical devices. In addition, the use of depolymerases in synergy with antibiotics has been proposed as a potent tool against *Klebsiella* sp. [21,22].

The high specificity of *Klebsiella* phages (and many other phages) is a double-edged sword. On the one hand, specificity allows precise targeting of a given bacterial pathogen and helps avoiding dysbiosis by exerting a minimal impact on the normal microbiota, which makes phages a suitable tool for personalized medicine [23]. Additionally, phages can be used as a bacterial typing tool for diagnosis, and can be genetically modified to be used as biosensors or in bioimaging [24]. On the other hand, specificity makes treatment options less obvious and hence requires a considerable effort in terms of characterizing and exploiting natural phage diversity. Here, we reported four phages active against a given bacterial capsular type, but further work will be needed to cover the extensive capsular diversity shown by *Klebsiella* sp. Phage ubiquity and abundance in widely different environments suggests that this goal is attainable.

## 4. Materials and Methods

### 4.1. Bacterial Isolates

Twenty-four clinical isolates of carbapenem-resistant *K. pneumoniae* were used (Table S2). These strains belong to a clinical collection from hospitals of the Comunidad Valenciana (Spain). Additionally, *NTUH-K2044* [25] and the *K. pneumoniae K22* reference strain from the Statens Serum Institute (Copenhagen, Denmark) were included.

### 4.2. Phage Isolation and Amplification

Environmental samples near sewage water plants in Valencia (Spain) from soil and water were tested against a clinical isolate of *K. pneumoniae* capsular type K22 (isolate *1210*). Briefly, 50 mL of water or 50 g of soil were placed in 100 mL tubes and kept at room temperature until use. Liquid samples were treated directly, and soil samples were soaked in Luria-Bertani (LB) broth. After vortexing or mixing vigorously, samples were centrifuged (13,000× *g*, 3 min) and supernatants were filtered (0.22 μm) twice per sample. Then, 1 mL of the filtered sample was added to 1 mL of bacteria in stationary phase (from an overnight culture), and poured onto Petri dishes using the soft agar overlay method. Dishes were incubated at 37 °C for 24 h to allow phage plaques to develop. Isolated plaques were picked by micropipette aspiration using filter tips and kept at −80 °C.

Verification of candidate plaques was done by re-infecting the same bacterial strain and by titration using serial dilutions. Verified plaques (*πVLC1-4*) were purified by the triple plaque assay method, which consists of doing three serial plaque assay/plaque picking steps. Determination of plaque diameters, plaque morphology, whether plaques were clear or turbid, and other features such as edges, halos, etc., was also done. Selected plaques were amplified in liquid culture in the same bacterial strain in which they were isolated to obtain high-titer lysates. To accomplish this, triple-purified plaques were serially diluted 1/10 in LB and used to infect a log-phase bacterial culture (OD_600_ = 0.4). After lysis (typically 2–3 h post inoculation), bacteria were removed by centrifugation (13,000× *g*, 3 min, twice) and the supernatant was aliquoted and kept at −80 °C for further assays.

### 4.3. Electron Microscopy

High titer lysates (ca. 10^11^ PFU/mL) of each phage (*πVLC1-4*) were centrifuged at 16,000× *g* and supernatants were filtered through 0.22 µm to remove bacteria and cell debris. A drop from each clarified lysate was deposited onto a carbon-coated Formvar supported by a 300 mesh copper grid and air-dried for 30 min. Excess liquid was withdrawn with filter paper. Phages were negatively stained with 2% phosphotungstic acid and examined under an electron microscope Jeol JEM-1010.

### 4.4. DNA Isolation and Genome Sequencing

DNA was extracted from gradient-purified preparations by treating these preparations with DNAse I to remove non-encapsidated DNA, followed by extraction using a commercial kit (QIAamp Viral RNA Mini Kit, QIAGEN, Hilden, Germany). DNA was tagged for library preparation and sequenced with an Illumina MiSeq machine (250 paried-end reads). Reads were screened for contaminants prior to de novo assembly using Kraken 2 [26]. Bacterial contaminants were not found and raw reads were assembled with SPAdes v3.9.1 [27]. Contigs smaller than 1000 nucleotides were discarded, resulting in a single contig for each genome. PHASTER [28] and BLAST were used to determine the closest phage sequences. To check whether the genome assemblies were complete and collinear to other *Drulisvirus* sequences, all complete sequences of this genus were downloaded from Genbank and aligned with the novel *Klebsiella* phage genomes. Additionally, phages *LIMElight* and *VP93* were included as outgroups [15]. We used ProgressiveMAUVE [29] to obtain the multiple sequence alignment and MAUVE viewer [30] to assess synteny and genome conservation. Start and end positions of the new phage genomes were defined according to phage *NTUH-K2044-K1* (NC_025418) using a Perl script available at https://gist.github.com/leosanbu/xmfa2fas.pl. The complete genome sequences are available from Genbank (accession No. MN794000-MN794003).

### 4.5. Genome Annotation

Phages πVLC1-4 were annotated using PHANOTATE [31], Glimmer v.3.0 [32] and Prodigal [33]. The later are implemented in PHASTER [28] and Prokka v.1.14 [34], respectively. Gene callings and start and stop coordinates were compared with a custom script (comp_phannot.r) available at https://github.com/BBeamud/. Briefly, an ORF was called if at least two of the algorithms agreed or if it was called only by PHANOTATE with a score ≤−3. Prodigal was prioritized over Glimmer and PHASTER to assign start and end coordinates for CDSs as previously suggested [35]. Next, nucleotide sequences for each predicted ORF were extracted with seqtk subseq (https://github.com/lh3/seqtk) and used for functional annotation. A custom phage protein database was built with makeblastdb from the one available at http://millardlab.org/bioinformatics/bacteriophage-genomes/ (August 2019), which contains all non-redundant phage proteins deposited in Genbank and their annotation. BLASTX searches were performed with an e-value cutoff of 1 × 10^−5^. The best BLASTX hit of non-hypothetical proteins, if possible, was retrieved. Additionally, CDSs were searched against a local database constructed with depolymerase proteins included in Pires et al. [36]. If any significant similarity was found, InterProScan 5 [37] and HHpred [38] were used for further checking domains and protein structures.

Lastly, we searched for any indicator of temperate behaviour: mobile genetic elements, antibiotic resistance genes, virulence genes or any kind of bacterial gene. Local databases from ICEberg v.2.0 (http://db-mml.sjtu.edu.cn/ICEberg/), the Comprehensive Antibiotic Resistance Database (CARD, https://card.mcmaster.ca/), Virulence factors of Pathogenic Bacteria (VFDB, http://www.mgc.ac.cn/VFs/main.htm) and BacteriaDB (in-house database, 2016) were used for this. Bacteriophage genomes were screened for similarity with the previous databases using BLASTN with an e-value cutoff of 1 × 10^−5^.

### 4.6. Comparative Genomics

Whole-genome average nucleotide identity (ANI) values between the new *Klebsiella* phages and the other *Drulisvirus* and *Drulisvirus-like* (*VP93, LIMElight*) phages were estimated using pANIto (https://github.com/sanger-pathogens/panito). Conserved and unique genes among the *Drulisvirus* were obtained with Proteinortho v.6 [39] comparing the previously extracted nucleotide CDSs and the ones available at Genbank. Maximum likelihood (ML) phylogenies were constructed with 1000 fast bootstrap pseudo-replicates using the GTR + G + I substitution model in IQ-TREE v.1.6.5 [40].

### 4.7. Spot Test

Spot tests in top-agar semi-solidified media were performed to determine the host range of the novel phages. Drops of 1 µL of each phage were poured onto bacterial lawns of different clinical isolates and incubated at 37 °C for 24 h to allow plaques to develop.

### 4.8. Liquid Infection

We inoculated 10^5^ PFU of phage in bacterial cultures at their exponential growth phase (log phase) and incubated the cultures at 37 °C inside a plate reader (Multiskan) for 10 h to determine the strength of lysis based on time-lapse turbidity (OD) measurements every 10 min.

## Figures and Tables

**Table 1:** Environmental conditions and sampling points. PFU: plaque-forming units.

*Klebsiella* Phage	Source	Location	Temperature	Date	Titer after Amplification (PFU/mL)
*πVLC1*	Water	Near sewage water plant	20 °C	28-March-2019	3.0 × 10^11^
*πVLC2*	Water	Near sewage water plant	20 °C	28-March-2019	2.4 × 10^11^
*πVLC3*	Soil	Near sewage water plant	17 °C	23-April-2019	2.7 × 10^11^
*πVLC4*	Soil	Near sewage water plant	20 °C	28-March-2019	1.0 × 10^10^

**Table 2:** De novo assembly of the novel *K. pneumoniae* phage genomes.

*Klebsiella* Phage	Size (bp)	Average Sequencing Coverage	GC Content (%)	Number of CDS	Nucleotide Pairwise Identity (%)		
					*πVLC1*	*πVLC2*	*πVLC3*
*πVLC1*	43,411	902	53.89	58	-		
*πVLC2*	43,784	816	53.89	60	99.78	-	
*πVLC3*	43,351	309	53.96	56	94.60	94.43	-
*πVLC4*	44,656	883	53.75	62	94.41	94.23	93.47

## Supplementary Materials

Supplementary materials can be found at https://www.mdpi.com/1422-0067/21/2/425/s1. Table S1: Functional annotation of *Klebsiella* phages *πVLC1-4* and orthology results. Table S2: Clinical isolates of carbapenem-resistant *K. pneumoniae* strains used in this study.

## References

[1] Domingo-Calap P., Georgel P., Bahram S. (2016). Back to the future: Bacteriophages as promising therapeutic tools. HLA.

[2] Kortright K.E., Chan B.K., Koff J.L., Turner P.E. (2019). Phage therapy: A renewed approach to combat antibiotic-resistant bacteria. Cell Host Microbe.

[3] Founou R.C., Founou L.L., Essack S.Y. (2017). Clinical and economic impact of antibiotic resistance in developing countries: A systematic review and meta-analysis. PLoS ONE.

[4] Cabral J.P.S. (2010). Water microbiology. Bacterial pathogens and water. Int. J. Environ. Res. Public Health.

[5] Holt K.E., Wertheim H., Zadoks R.N., Baker S., Whitehouse C.A., Dance D., Jenney A., Connor T.R., Hsu L.Y., Severin J. (2015). Genomic analysis of diversity, population structure, virulence, and antimicrobial resistance in *Klebsiella pneumoniae*, an urgent threat to public health. Proc. Natl. Acad. Sci. USA.

[6] Wyres K.L., Holt K.E. (2016). *Klebsiella pneumoniae* population genomics and antimicrobial-resistant clones. Trends Microbiol..

[7] David S., Reuter S., Harris S.R., Glasner C., Feltwell T., Argimon S., Abudahab K., Goater R., Giani T., Errico G. (2019). Epidemic of carbapenem-resistant *Klebsiella pneumoniae* in Europe is driven by nosocomial spread. Nat. Microbiol..

[8] Wyres K.L., Wick R.R., Gorrie C., Jenney A., Follador R., Thomson N.R., Holt K.E. (2016). Identification of *Klebsiella* capsule synthesis loci from whole genome data. Microb. Genom..

[9] Majkowska-Skrobek G., Latka A., Berisio R., Squeglia F., Maciejewska B., Briers Y., Drulis-Kawa Z. (2018). Phage-borne depolymerases decrease *Klebsiella pneumoniae* resistance to innate defense mechanisms. Front. Microbiol..

[10] Pan Y.-J., Lin T.-L., Chen C.-C., Tsai Y.-T., Cheng Y.-H., Chen Y.-Y., Hsieh P.-F., Lin Y.-T., Wang J.-T. (2017). *Klebsiella* phage *ΦK64-1* encodes multiple depolymerases for multiple host capsular types. J. Virol..

[11] Sullivan M.J., Petty N.K., Beatson S.A. (2011). Easyfig: A genome comparison visualizer. Bioinformatics.

[12] Hughes K.A., Sutherland I.W., Clark J., Jones M.V. (1998). Bacteriophage and associated polysaccharide depolymerases—Novel tools for study of bacterial biofilms. J. Appl. Microbiol..

[13] Schmidt C. (2019). Phage therapy’s latest makeover. Nat. Biotechnol..

[14] Drulis-Kawa Z., Mackiewicz P., Kęsik-Szeloch A., Maciaszczyk-Dziubinska E., Weber-Dąbrowska B., Dorotkiewicz-Jach A., Augustyniak D., Majkowska-Skrobek G., Bocer T., Empel J. (2011). Isolation and characterisation of *KP34*—A novel *φKMV-like* bacteriophage for *Klebsiella pneumoniae*. Appl. Microbiol. Biotechnol..

[15] Eriksson H., Maciejewska B., Latka A., Majkowska-Skrobek G., Hellstrand M., Melefors Ö., Wang J.-T., Kropinski A.M., Drulis-Kawa Z., Nilsson A.S. (2015). A suggested new bacteriophage genus, “*Kp34likevirus*”, within the *Autographivirinae* subfamily of *Podoviridae*. Viruses.

[16] Lin T.-L., Hsieh P.-F., Huang Y.-T., Lee W.-C., Tsai Y.-T., Su P.-A., Pan Y.-J., Hsu C.-R., Wu M.-C., Wang J.-T. (2014). Isolation of a bacteriophage and its depolymerase specific for K1 capsule of *Klebsiella pneumoniae*: Implication in typing and treatment. J. Infect. Dis..

[17] Solovieva E.V., Myakinina V.P., Kislichkina A.A., Krasilnikova V.M., Verevkin V.V., Mochalov V.V., Lev A.I., Fursova N.K., Volozhantsev N.V. (2018). Comparative genome analysis of novel Podoviruses lytic for hypermucoviscous *Klebsiella pneumoniae* of K1, K2, and K57 capsular types. Virus Res..

[18] North O.I., Sakai K., Yamashita E., Nakagawa A., Iwazaki T., Büttner C.R., Takeda S., Davidson A.R. (2019). Phage tail fibre assembly proteins employ a modular structure to drive the correct folding of diverse fibres. Nat. Microbiol..

[19] Fernandes S., São-José C. (2018). Enzymes and mechanisms employed by tailed bacteriophages to breach the bacterial cell barriers. Viruses.

[20] Dams D., Briers Y., Labrou N. (2019). Enzybiotics: Enzyme-based antibacterials as therapeutics. Therapeutic Enzymes: Function and Clinical Implications.

[21] Bansal S., Harjai K., Chhibber S. (2014). Depolymerase improves gentamicin efficacy during *Klebsiella pneumoniae* induced murine infection. BMC Infect. Dis..

[22] Domingo-Calap P., Delgado-Martínez J. (2018). Bacteriophages: Protagonists of a post-antibiotic era. Antibiotics.

[23] Hsu B.B., Gibson T.E., Yeliseyev V., Liu Q., Lyon L., Bry L., Silver P.A., Gerber G.K. (2019). Dynamic modulation of the gut microbiota and metabolome by bacteriophages in a mouse model. Cell Host Microbe.

[24] Domingo-Calap P. (2018). Phages as promising biomedical tools. Biomed. J. Sci. Tech. Res..

[25] Wu K.-M., Li L.-H., Yan J.-J., Tsao N., Liao T.-L., Tsai H.-C., Fung C.-P., Chen H.-J., Liu Y.-M., Wang J.-T. (2009). Genome sequencing and comparative analysis of *Klebsiella pneumoniae NTUH-K2044*, a strain causing liver abscess and meningitis. J. Bacteriol..

[26] Wood D.E., Lu J., Langmead B. (2019). Improved metagenomic analysis with Kraken 2. Genome Biol..

[27] Bankevich A., Nurk S., Antipov D., Gurevich A.A., Dvorkin M., Kulikov A.S., Lesin V.M., Nikolenko S.I., Pham S., Prjibelski A.D. (2012). SPAdes: A new genome assembly algorithm and its applications to single-cell sequencing. J. Comput. Biol..

[28] Arndt D., Grant J.R., Marcu A., Sajed T., Pon A., Liang Y., Wishart D.S. (2016). PHASTER: A better, faster version of the PHAST phage search tool. Nucleic Acids Res..

[29] Darling A.E., Mau B., Perna N.T. (2010). ProgressiveMauve: Multiple genome alignment with gene gain, loss and rearrangement. PLoS ONE.

[30] Darling A.C.E., Mau B., Blattner F.R., Perna N.T. (2004). Mauve: Multiple alignment of conserved genomic sequence with rearrangements. Genome Res..

[31] McNair K., Zhou C., Dinsdale E.A., Souza B., Edwards R.A. (2019). PHANOTATE: A novel approach to gene identification in phage genomes. Bioinformatics.

[32] Delcher A.L., Harmon D., Kasif S., White O., Salzberg S.L. (1999). Improved microbial gene identification with GLIMMER. Nucleic Acids Res..

[33] Hyatt D., Chen G.-L., Locascio P.F., Land M.L., Larimer F.W., Hauser L.J. (2010). Prodigal: Prokaryotic gene recognition and translation initiation site identification. BMC Bioinform..

[34] Seemann T. (2014). Prokka: Rapid prokaryotic genome annotation. Bioinformatics.

[35] Salisbury A., Tsourkas P.K. (2019). A method for improving the accuracy and efficiency of bacteriophage genome annotation. Int. J. Mol. Sci..

[36] Pires D.P., Oliveira H., Melo L.D.R., Sillankorva S., Azeredo J. (2016). Bacteriophage-encoded depolymerases: Their diversity and biotechnological applications. Appl. Microbiol. Biotechnol..

[37] Jones P., Binns D., Chang H.-Y., Fraser M., Li W., McAnulla C., McWilliam H., Maslen J., Mitchell A., Nuka G. (2014). InterProScan 5: Genome-scale protein function classification. Bioinformatics.

[38] Zimmermann L., Stephens A., Nam S.-Z., Rau D., Kübler J., Lozajic M., Gabler F., Söding J., Lupas A.N., Alva V. (2018). A completely reimplemented MPI bioinformatics toolkit with a new HHpred Server at its core. J. Mol. Biol..

[39] Lechner M., Findeiß S., Steiner L., Marz M., Stadler P.F., Prohaska S.J. (2011). Proteinortho: Detection of (Co-)orthologs in large-scale analysis. BMC Bioinform..

[40] Nguyen L.-T., Schmidt H.A., von Haeseler A., Minh B.Q. (2015). IQ-TREE: A fast and effective stochastic algorithm for estimating maximum-likelihood phylogenies. Mol. Biol. Evol..

