# Supplementary material for: Isolation of Four Lytic Phages Infecting Klebsiella pneumoniae K22 Clinical Isolates from Spain
Source: Int J Mol Sci. 2020 Jan 9;21(2):425. doi: 10.3390/ijms21020425 (PMC7013548; doi:10.3390/ijms21020425)
Supplement: Supplementary file 1 [file ijms-21-00425-s001.zip › ijms-674612-supplementary/Supplementary tables/Table S1.docx]

**Table S1.** Functional annotation of *Klebsiella* phages πVLC1-4 and orthology results. *: absent. Red bold: exclusive putative genes of this group compared to other *Drulisvirus* phages.

| **ORF** | **ORF position** | | | | **Predicted function** |
| --- | --- | --- | --- | --- | --- |
|  | πVLC1 | πVLC2 | πVLC3 | πVLC4 |  |
| 1 | * | * | * | 596..691 | Hypothetical protein |
| 2 | * | * | * | 661..1053 | HNN endonuclease |
| 3 | 1209..1427 | 1199..1417 | 1304..1519 | 1817..2032 | Hypothetical protein |
| 4 | 1430..1534 | 1420..1524 | * | * | Hypothetical protein |
| 5 | 1610..2185 | 1600..2175 | 1591..2166 | 2196..2771 | Hypothetical protein |
| 6 | 2182..2313 | 2172..2303 | 2163..2294 | 2768..2899 | Hypothetical protein |
| 7 | 2375..2524 | 2365..2514 | 2356..2505 | * | Hypothetical protein |
| 8 | * | * | * | 2936..3172 | Hypothetical protein |
| 9 | 2600..2821 | 2590..2811 | 2581..2802 | 3174..3395 | DNA topoisomerase II large subunit |
| 10 | 2814..3077 | 2804..3067 | 2795..3058 | 3388..3651 | Hypothetical protein |
| 11 | * | * | * | 3663..3902 | Hypothetical protein |
| 12 | 3086..3265 | 3076..3255 | 3067..3246 | 3899..4084 | Hypothetical protein |
| 13 | * | 3252..3470 | * | * | Hypothetical protein |
| 14 | * | 3458..3946 | * | * | HNH endonuclease |
| 15 | * | 3943..4287 | * | * | Hypothetical protein |
| 16 | 3262..3465 | * | 3243..3446 | 4081..4284 | Hypothetical protein |
| 17 | 3556..5526 | 4337..6010 | 3537..5504 | 4375..6345 | Hypothetical protein |
| 18 | 5526..6572 | 6010..7056 | 5504..6550 | 6345..7391 | Peptidase |
| 19 | 6575..7018 | 7059..7502 | * | 7394..7837 | Hypothetical protein |
| 20 | * | * | 6581..7060 | * | HNH endonuclease |
| 21 | 7015..7215 | 7499..7699 | * | * | Hypothetical protein |
| 22 | * | * | * | 7834..8052 | Hypothetical protein |
| 23 | 7215..8024 | 7699..8508 | 7017..7820 | 8055..8864 | DNA primase |
| 24 | 7997..8137 | 8481..8621 | * | 8837..8977 | Eaa protein |
| 25 | * | * | 7813..8016 | * | Hypothetical protein |
| 26 | * | * | 8018..8206 | * | Hypothetical protein |
| 27 | * | * | 8206..8346 | * | Hypothetical protein |
| 28 | 8125..9405 | 8609..9889 | 8349..9614 | 8965..10245 | DNA helicase |
| 29 | 9456..9614 | 9940..10098 | * | 10296..10454 | Membrane-associated initiation of head vertex |
| 30 | 9607..9762 | 10091..10246 | 9816..9971 | 10447..10602 | Hypothetical protein |
| 31 | **9759..10130** | **10243..10614** | **9968..10339** | **10599..10970** | Hypothetical protein |
| 32 | * | * | 10314..10769 | 10945..11400 | HNH endonuclease |
| 33 | 10105..12474 | 10589..12958 | 10766..13108 | 11397..13757 | DNA polymerase |
| 34 | 12471..12692 | 12955..13176 | 13105..13326 | 13754..13975 | Hypothetical protein |
| 35 | 12854..13891 | 13338..14375 | 13489..14466 | 14137..15117 | Phospohoesterase |
| 36 | 13939..14400 | 14423..14884 | * | * | HNH endonuclease |
| 37 | * | * | * | 15131..15325 | Hypothetical protein |
| 38 | 14406..15239 | 14890..15723 | 14518..15351 | 15381..16214 | Large tegument protein |
| 39 | 15292..15546 | 15776..16030 | * | 16267..16521 | Hypothetical protein |
| 40 | * | * | 15404..15547 | * | Hypothetical protein |
| 41 | * | * | * | 16522..16800 | Hypothetical protein |
| 42 | 15550..15921 | 16034..16405 | 15561..15935 | 16800..17174 | Hypothetical protein |
| 43 | 15921..16082 | 16402..16566 | 15935..16096 | 17174..17335 | Hypothetical protein |
| 44 | 16085..16243 | 16569..16727 | 16099..16257 | 17338..17496 | Hypothetical protein |
| 45 | 16243..17211 | 16727..17695 | 16257..17225 | 17496..18464 | 5'-3' Exonuclease |
| 46 | 17168..17368 | 17652..17852 | 17182..17382 | 18421..18621 | beta-galactosidase |
| 47 | * | * | * | 18615..19061 | HNH endonuclease |
| 48 | 17362..17811 | 17846..18295 | 17376..17825 | * | HNH endonuclease |
| 49 | 17793..18215 | 18277..18699 | 17807..18229 | * | Endonuclease |
| 50 | * | * | * | 19043..19465 | Endonuclease |
| 51 | 18212..18676 | 18696..19160 | 18226..18690 | 19462..19926 | Nucleotide kinase |
| 52 | 18663..18779 | 19174..19263 | 18677..18793 | 19913..20029 | Hypothetical protein |
| 53 | 18818..21286 | 19302..21770 | 18832..21300 | 20057..22525 | RNA polymerase |
| 54 | 21310..21750 | 21794..22234 | 21324..21764 | 22549..22989 | Membrane-associated initiation of head vertex; |
| 55 | 21747..22010 | 22231..22494 | 21761..22024 | 22986..23249 | Hypothetical protein |
| 56 | 22020..23615 | 22504..24099 | 22034..23629 | 23259..24854 | Head-tail connector protein |
| 57 | 23630..24472 | 24114..24956 | 23644..24486 | 24869..25711 | Scaffolding protein |
| 58 | 24498..25517 | 24982..26001 | 24512..25531 | 25737..26756 | Capsid protein |
| 59 | 25529..25711 | 26013..26195 | 25543..25725 | 26768..26950 | Hypothetical protein |
| 60 | 25798..26265 | 26298..26765 | 25811..26278 | 27039..27506 | Hypothetical protein |
| 61 | 26315..26818 | 26815..27318 | 26328..26831 | 27556..28059 | Tail tubular protein A |
| 62 | **26828..27316** | **27328..27816** | **26841..27329** | **28069..28557** | HNH endonuclease |
| 63 | 27411..29669 | 27911..30169 | 27424..29682 | 28652..30910 | Tail tubular protein B |
| 64 | 29671..30258 | 30171..30758 | 29684..30271 | 30912..31499 | Internal virion protein B |
| 65 | 30276..32960 | 30776..33460 | 30289..32973 | 31517..34201 | Hypothetical protein |
| 66 | 33011..36709 | 33511..37059 | 33024..36722 | 34252..37950 | Internal core protein |
| 67 | * | 37065..37208 | * | * | Internal core protein |
| 68 | **36711..38945** | **37210..39552** | **36724..39066** | **37952..40186** | Tail fiber protein |
| 69 | 38938..39180 | * | * | 40179..40421 | Tail fiber protein |
| 70 | **39185..39349** | **39557..39721** | **39071..39235** | **40426..40590** | Hypothetical protein |
| 71 | 39364..39666 | 39736..40038 | 39250..39552 | 40605..40907 | DNA maturase A |
| 72 | 39666..41522 | 40038..41894 | 39552..41408 | 40907..42763 | DNA maturase B |
| 73 | 41522..41845 | 41894..42217 | * | 42763..43086 | Hypothetical protein |
| 74 | * | * | 41408..41782 | * | Hypothetical protein |
| 75 | 41857..42039 | 42229..42411 | 41794..41976 | 43098..43280 | Hypothetical protein |
| 76 | 42039..42443 | 42411..42815 | 41976..42380 | 43280..43684 | Spanin |
| 77 | 42436..42687 | 42808..43059 | 42373..42624 | 43677..43928 | Holin |
| 78 | 42671..43279 | 43043..43651 | 42608..43216 | 43912..44520 | Endolysin |
