# Supplementary material for: Isolation of Four Lytic Phages Infecting Klebsiella pneumoniae K22 Clinical Isolates from Spain
Source: Int J Mol Sci. 2020 Jan 9;21(2):425. doi: 10.3390/ijms21020425 (PMC7013548; doi:10.3390/ijms21020425)
Supplement: Supplementary file 1 [file ijms-21-00425-s001.zip › ijms-674612-supplementary/Supplementary tables/Table S2.docx]

**Table S2.** Clinical isolates of carbapenem-resistant *K. pneumoniae* strains used in this study. La Fe: Hospital Universitario La Fe (Valencia, Spain). HGV: Hospital General de Valencia (Valencia, Spain). HGUE: Hospital General Universitario de Elche (Elche, Spain). NA: not available.

| ***K. pneumoniae* strain** | **K-locus** | **Hospital** |
| --- | --- | --- |
| 1210 | 22 | La Fe |
| 2.3 | NA | HGUE |
| NTUH | 1 | Taiwan |
| x4 | 60 | HGV |
| x5 | 126 | HGV |
| x8 | 1 | HGV |
| x9 | 10 | HGV |
| x10 | 25 | HGV |
| x11 | 140 | HGV |
| x12 | 39 | HGV |
| x13 | 140 | HGV |
| x14 | 157 | HGV |
| x17 | 15 | HGV |
| x18 | 30 | HGV |
| x20 | 111 | HGV |
| x22 | 61 | HGV |
| x24 | 112 | HGV |
| x29 | 139 | HGV |
| x31 | 123 | HGV |
| x32 | 125 | HGV |
| x33 | 14 | HGV |
| x34 | 16 | HGV |
| x37 | 20 | HGV |
| x38 | 128 | HGV |
